# Chronic Wasting Disease in Free-Ranging Wisconsin White-Tailed Deer

**DOI:** 10.3201/eid0905.020721

**Published:** 2003-05

**Authors:** Damien O. Joly, Christine A. Ribic, Julie A. Langenberg, Kerry Beheler, Carl A. Batha, Brian J. Dhuey, Robert E. Rolley, Gerald Bartelt, Timothy R. van Deelen, Michael D. Samuel

**Affiliations:** * United States Geological Society-Wisconsin Cooperative Wildlife Research Unit, University of Wisconsin-Madison, Madison, Wisconsin, USA; † Wisconsin Department of Natural Resources, Madison, Wisconsin, USA; ‡ Wisconsin Department of Natural Resources, Monona, Wisconsin, USA; § Wisconsin Department of Natural Resources, Rhinelander, Wisconsin, USA; ¶ United States Geological Society-National Wildlife Health Center, Madison, Wisconsin, USA

**Keywords:** White-tailed Deer, prion diseases, Wisconsin, dispatch

Three White-Tailed Deer shot within 5 km during the 2001 hunting season in Wisconsin tested positive for chronic wasting disease, a prion disease of cervids. Subsequent sampling within 18 km showed a 3% prevalence (n=476). This prevalence indicates a significant range extension for chronic wasting disease into the eastern United States.

Chronic wasting disease (CWD) is degenerative and usually considered to be fatal in White-tailed Deer (*Odocoileus virginianus*), Mule Deer (*O. hemionus*), and Elk (*Cervus elaphus*) associated with the presence of transmissible protease-resistant prion proteins (PrP^cwd^) (*1*,*2*). Although the transmission route of PrP^cwd^ is unknown, it may be transmitted in deer and elk by direct or indirect contact from the environment (*1*,*2*). In experiments, clinical signs have appeared as early as 15 months after exposure (*1*) and include weight loss, anorexia, repetitive behaviors, hyperesthesia, and intractability. Signs progress to severe emaciation, extreme behavioral changes, excessive salivation, tremors, and mild ataxia (*1*,*2*). CWD was first recognized in captive Mule Deer in Colorado (*3*) and subsequently described in the free-ranging cervid populations of Colorado and Wyoming (*1*); prevalence in these disease-endemic areas varies spatially and among the three sympatric cervid species (*4*). Before its discovery in Wisconsin, CWD was detected in captive cervid farms in Colorado, Nebraska, South Dakota, Oklahoma, Kansas, Montana (USA), as well as Alberta, Saskatchewan (Canada), and South Korea (*1*). Apart from the contiguous areas of Colorado, Wyoming, and Nebraska, CWD had previously only been detected in two free-ranging Mule Deer from Saskatchewan, one Mule Deer from South Dakota, and in a number of Mule Deer from the western slopes region of Colorado (*1*). Previously, no cases of CWD were reported east of the Mississippi River; however, subsequent to our research, CWD-positive cervids were found in Minnesota (captive elk), and Wisconsin (captive White-tailed Deer). Further, west of Mississippi, CWD-positive Mule deer have been found in New Mexico and Utah, free-ranging White-tailed Deer in Saskatchewan, Canada, and captive White-tailed Deer in Alberta, Canada. On CWD-positive free-ranging Mule Deer was discovered in New Mexico.

## The Study

In autumn of 1999 and 2000, the Wisconsin Department of Natural Resources (WDNR) submitted to the National Veterinary Services Laboratories (NVSL) (Ames, Iowa) brain material (obex) from 657 hunter-killed White-tailed Deer registered at hunter check stations across the state. None came from the study area we describe. Samples were tested for CWD prion by immunohistochemistry (IHC) (*5*). Prion was not detected in any samples. However, 3 of 445 White-tailed Deer shot in autumn of 2001 were positive for CWD. These deer were males, 2.5 years of age, and were shot within 5 km in south-central Wisconsin. WDNR subsequently conducted a sampling program to assess the distribution and prevalence of CWD in the vicinity of these three positive deer. We report the results of this sampling program.

Samples were collected from 500 adult (>1 year of age) White-tailed Deer within an approximate 18-km radius, and all samples were tested for CWD. Deer were submitted by hunters who were issued scientific collection permits to collect deer at roadside after a vehicular collision or collected by hunters for WDNR or U.S. Department of Agriculture. Data from collected deer included the geographic location based on the Wisconsin Public Land Survey System (township-range-section), sex, and age (estimated by using tooth eruption and tooth wear patterns). Location of kill was indicated on a map by hunters during interviews by DNR staff (*6*). Samples of brain stem (obex) and retropharyngeal lymphatic tissue were fixed in 10% of buffere formalin and submitted to NVSL for testing using IHC. We considered a deer to be CWD positive if either obex or retropharyngeal samples were IHC positive (*1*).

We used the spatial scan statistic provided by Kulldorff and Nagarwalla (*7*) (program SaTScan available from: URL: http://www3.cancer.gov/prevention/bb/satscan.html) to assess the presence and location of CWD clusters within the surveillance area. Location data were collected to the survey unit “section” (approximately 2.6 km^2^). We pooled locations into 4X4 section quadrants for analysis to compensate for sections from which no deer were collected. In a separate analysis, sex and age were assessed as predictors of CWD status by using logistic regression (function glm in program R v. 1.5.0; available from: URL: http://www.r-project.org1) (*8*). Model selection uncertainty was incorporated into the odds ratio (OR) estimates by using model averaging (9).

## Results and Discussion

From March 2 to April 9, 2002, samples were collected from 505 deer; however, 29 deer were not included in the analysis because of sample autolysis, inappropriate tissue submission, or lack of availability of appropriate tissues (e.g., deer with no intact cranium or those shot in the head). Of the remaining 476 deer (87 males, 386 females, and 3 for which sex was not recorded), 15 (3.2%; 95% confidence limit [CI] 1.7% to 5.1%) were IHC positive, 11 in both obex and retropharyngeal lymph node samples and 4 from lymph nodes only. We inferred that deer that were only lymph node positive were in the earlier states of infection (*1*). Estimated prevalence varied spatially within the surveillance area. A cluster of higher than expected prevalence was detected in the north-central region of the sampling area (prevalence 9.4%; 95% CI 5.0% to 16.0%; p=0.003; n=127) (Figure).

**Figure:**
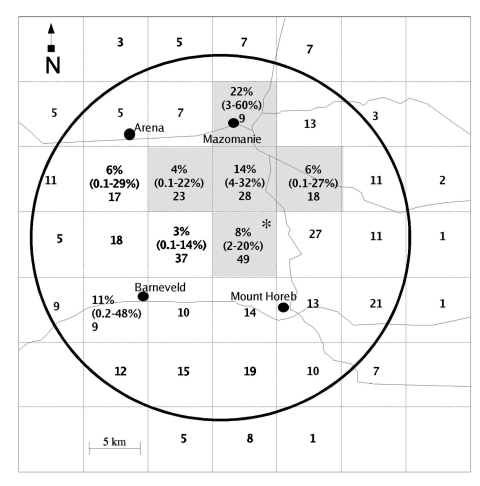
Spatial distribution of chronic wasting disease in White-tailed Deer sampled in Wisconsin (February–April 2002). Locations for sampled deer were recorded by using the Wisconsin Public Land Survey System (township-range-section); analysis was conducted on pooled 4X4 sections (41 km^2^), as indicated by the dashed grid lines. Prevalence, 95% confidence limits (CI), and sample size for each quadrant are indicated, as well as sample size only for quadrants in which positive deer were not detected. A cluster of higher than expected prevalence was detected in the north-central region of the sampling area indicated by shading (prevalence 9.4%, 95% CI 5.0% to 16.0%, n=127). The asterisk indicates the quadrant in which the three initial positive deer were found. The circle represents the targeted surveillance area.

Prevalence did not vary by sex (males: 3.4%, 95% CI 0.1% to 9.7%, n=87; females: 3.1%, 95% CI 1.6% to 5.3%, n=386; male vs. female OR 1.1, 95% CI 0.56 to 2.19), a pattern consistent with Mule Deer sampled in Colorado and Wyoming (*4*). Increasing prevalence with age was suggested, although we could not distinguish whether the OR differed from 1 (OR 1.13, 95% CI 0.93 to 1.39). We had a small sample (n=32) of older animals (>5 years of age), which weakened our ability to detect an increase in prevalence with age statistically. Miller et al. (*4*) found that CWD prevalence increased with age in male Mule Deer and then abruptly declined in older age classes. We did not have a sufficient sample size to evaluate a sex difference in prevalence by age.

The known range of CWD was extended by its detection in Wisconsin, which is the first report of the disease east of the Mississippi River. Although we do not know how the free-ranging deer population of Wisconsin became affected by CWD, the most commonly suggested hypothesis is that CWD in Wisconsin may have emerged through importing of an affected cervid. The current enzootic of CWD in free-ranging deer and elk is paralleled by an enzootic in the captive cervid industry, and the relationship between CWD-affected elk farms and recent (2000–2002) diagnoses of CWD in free-ranging deer in Nebraska, South Dakota, and Saskatchewan remains under investigation (*1*). Elk were imported to Wisconsin from CWD-affected herds in Colorado during the 1990s, and recently (September and October 2002) captive White-tailed Deer were found to be positive on two separate farms in central and southern Wisconsin (*10*). Furthermore, during epidemiologic investigations of these positive farms, we discovered that deer had escaped in March 2002 from one of these farms, one of which was later shot and found to be CWD positive (*9*). We stress that these positive captive deer are likely not the source of CWD in this free-ranging White-tailed Deer outbreak because of the captive deer’s distance from the area where the free-ranging deer are (approximately 130 km). No direct evidence exists that CWD came to Wisconsin by the captive cervid industry. However, further investigation on possible links between CWD cases in captive and free-ranging cervids in Wisconsin is ongoing.

## Conclusions

The state of Wisconsin is undertaking an integrated research, surveillance, and management program to determine the distribution of CWD in Wisconsin free-ranging deer population and eventually eliminating the disease from the known affected area of south-central Wisconsin (*10*,*11*). In March 2003, a total of 39,636 deer had been sampled statewide for CWD as part of this surveillance and management program (data are available from: URL: http://www.dnr.state.wi.us/org/land/wildlife/whealth/issues/CWD/). Computer simulation of CWD dynamics in western cervid populations (*12*) indicated that CWD could severely reduce deer numbers. Disease transmission may occur at a greater rate and consequently have a larger impact on the population in the eastern United States, where White-tailed Deer densities are typically an order of magnitude larger than western deer and elk populations (e.g., deer densities in the CWD-affected area are estimated to be currently >150 deer per km^2^) (WDNR, unpub. data). Deer and deer-related activities, such as hunting, wildlife viewing, and other social factors, are an important component of the Wisconsin culture and economy (approximately $1 billion/year) (*13*), prompting an aggressive research and management strategy to combat CWD in Wisconsin’s free-ranging deer population.
